# The updated landscape of tumor microenvironment and drug repurposing

**DOI:** 10.1038/s41392-020-00280-x

**Published:** 2020-08-25

**Authors:** Ming-Zhu Jin, Wei-Lin Jin

**Affiliations:** 1 Institute of Nano Biomedicine and Engineering, Shanghai Engineering Center for Intelligent Diagnosis and Treatment Instrument, Department of Instrument Science and Engineering, Key Laboratory for Thin Film and Microfabrication Technology of Ministry of Education, School of Electronic Information and Electronic Engineering, Shanghai Jiao Tong University, Shanghai, 200240 P. R. China; 2 Shanghai Jiao Tong University School of Medicine, Shanghai, 200025 P. R. China

**Keywords:** Cancer microenvironment, Cancer microenvironment, Translational research

## Abstract

Accumulating evidence shows that cellular and acellular components in tumor microenvironment (TME) can reprogram tumor initiation, growth, invasion, metastasis, and response to therapies. Cancer research and treatment have switched from a cancer-centric model to a TME-centric one, considering the increasing significance of TME in cancer biology. Nonetheless, the clinical efficacy of therapeutic strategies targeting TME, especially the specific cells or pathways of TME, remains unsatisfactory. Classifying the chemopathological characteristics of TME and crosstalk among one another can greatly benefit further studies exploring effective treating methods. Herein, we present an updated image of TME with emphasis on hypoxic niche, immune microenvironment, metabolism microenvironment, acidic niche, innervated niche, and mechanical microenvironment. We then summarize conventional drugs including aspirin, celecoxib, β-adrenergic antagonist, metformin, and statin in new antitumor application. These drugs are considered as viable candidates for combination therapy due to their antitumor activity and extensive use in clinical practice. We also provide our outlook on directions and potential applications of TME theory. This review depicts a comprehensive and vivid landscape of TME from biology to treatment.

## Persistent updating concept of TME

The concept of tumor microenvironment (TME) dates back to 1863 when Virchow proposed the relationship between inflammation and cancer and 1889 upon the emergence of Pagets’s theory of “seed and soil”^1–3^. In 2011, Hanahan and Weinberg enhanced their understanding of cancer on the basis of the great progress made over the past decade and expanded their proposed hallmarks of cancer from six to ten. They also recognized the emerging participation of TME in cancer development.^4^

Cancer is a complicated disease that metastasizes through vessels, lymph nodes, or transcoelomic seeding, is innervated by nerves, and involves the participation of the whole organism. Thus, a concern exists on whether TME is sufficiently comprehensive to reflect true situations and serve as an effective target for cancer treatment.^5^ Laplane et al.^5,6^ introduced a term called tumor organismal environment (TOE), representing microenvironments that are distant from the lesions of cancer but can affect its development. Considering the lack of consensus in defining TME combined with the fact that the tumor environment (TE) of different locations may differ greatly, they also divided TE into six layers, including tumor cell to tumor-cell environment (TCTCE), niche, confined TE, proximal TE, peripheral TE, and TOE.^6^

**Fig. 1:**
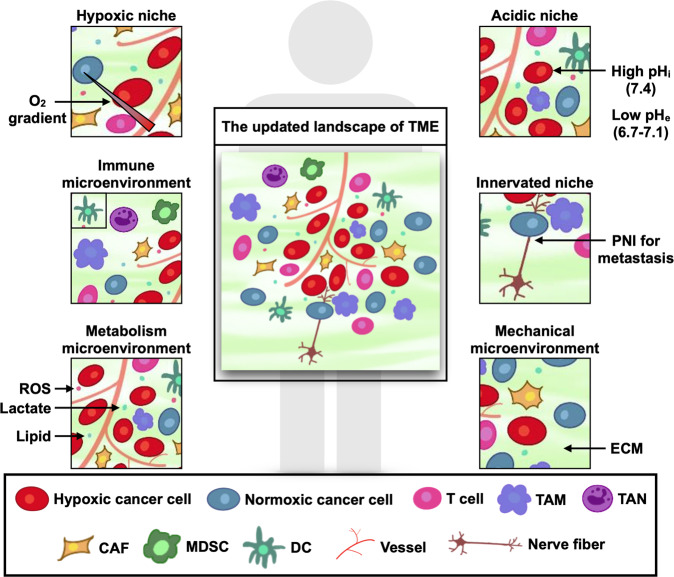
The updated landscape of tumor microenvironment (TME). TME comprises cancer cells, stromal cells, blood vessels, nerve fibers, extracellular matrix, and associated acellular components. TME is home for cancer cells and serves as a bridge connecting cancer with the whole organism. TME can be classified into six specialized microenvironments, namely, hypoxic niche, immune microenvironment, metabolism microenvironment, acidic niche, innervated niche, and mechanical microenvironment

The conventional concept of TME excludes peripheral TE with distally located lymphatic tissues and TOE, which is even more distant and comprises microbiota or some exosomes. TME comprises nonmalignant cells, vessels, lymphoid organs or lymph nodes, nerves, intercellular components, and metabolites located at the center, margin, or within the vicinity of the tumor lesion (Fig. 1). Classical theory suggests that the oncogenic mutations of malignant cells cause the initiation of cancer. Subsequently, the surrounding non-transformed cells are recruited and adapted, accompanied by the release of diverse intercellular communicators, including cytokines, chemokines, and vesicles. The consequences are TME formation and close interaction with cancer cells.^7^ Some studies have shown that chronic inflammation or wound-healing process in abnormal microenvironment activates oncogenic signaling and promotes tumorigenesis.^8–11^

TME is shaped and trained by cancer cells to assist the development of cancer hallmarks, respond to intrinsic or extrinsic stress, stimulation and treatment, and ultimately assist these cells’ survival and migration in an organism. Compared with a whole TME, a specialized microenvironment seems to be a better target for cancer treatment. Hypoxia and immunosuppression are two properties of cancer.^4^ Hypoxia was viewed as a hallmark of TME as early as 1955, whereas immune microenvironment was recognized in 1975 when tumor-infiltrating lymphocytes (TILs) were identified.^2^ Hypoxic niche and immune microenvironment are regarded as two vital specialized microenvironments that can reprogram cancer biology in various aspects and serve as a potential target for cancer therapy, especially targeted therapy and immunotherapy.^12–17^ Many studies have confirmed that metabolism microenvironment,^18–21^ acidic niche,^22–24^ innervated niche in TME^25–31^ and mechanical niche^32–37^ remarkably affect cancer development. However, these specialized microenvironments are arranged in a crisscross pattern, suggesting the necessity to recognize the function of each microenvironment and the crosstalk among one another (Fig. 1). The latent capacity of combination therapy cannot be ignored as well.

Notably, TME is heterogeneous and can be considered as a double-edged sword.^38–41^ Specially, TME at an early stage tends to exert an antitumor effect.^2,42^ This review primarily focuses on how specialized TMEs assist the development of cancer hallmarks.

## Hallmarks of TME

### Hypoxic niche: a constant and heterogeneous TME

In 2019, Kaelin, Ratcliffe, and Semenza were awarded the Nobel Prize in Physiology or Medicine for their work in cellular sensation and adaptation to oxygen. In response to normoxia-hypoxia transition, cells rely largely on the elevation of hypoxia-inducible factors (HIF) and HIF signaling to adapt to a low-oxygen condition.^43,44^ In cancer, intratumoral hypoxia is induced by the outgrowth of cancer cells and the unmatched angiogenesis and oxygen supply, accompanied by the altered metabolism level of cancer cells. Hypoxia is a common, constant, and complex condition for malignant and stromal cells. Intratumoral hypoxia is often correlated with poor prognosis and cancer progression.^13^ Hypoxia can exist in cancer cells and its microenvironment, subsequently reprogramming cancer biology in various aspects, including cancer progression, stemness, and dormancy, as well as redox adaptation, intercellular communication, and therapeutic resistance (reviewed by ref. ^13^). Hypoxia activates vascular endothelial cells, upregulates vascular endothelial growth factor (VEGF) transcription, and stimulates excessive angiogenesis, thereby affecting TME and therapeutic efficacy.^45–47^

Buffa et al.^48^ listed the 15 top-ranked hypoxia-associated genes, namely, VEGFA, SLC2A1, PGAM1, ENO1, LDHA, TP11, P4HA1, MRPS1, CDKN3, ADM, NDRG1, TUBB6, ALDOA, MIF, and ACOT7, which are collectively considered to be hypoxia signature (Buffa signature) to assess hypoxia status. Bhandari et al.^49^ studied 1188 cancers from 27 cancer types, including solid cancer and hematologic malignancies, to investigate hypoxia in cancer. They subjected 2658 cancers from 38 cancer types to whole-genome sequencing. The pan-cancer landscape reveals that hypoxia possesses intra- and inter-heterogeneity among different cancer types and different patients with the same type.^49^ For example, lung and cervical squamous cell carcinoma are regarded as the most hypoxic cancer types, whereas chronic lymphocytic leukemia and thyroid adenocarcinoma have the lowest hypoxia scores; biliary adenocarcinoma, lymphoid B-cell non-Hodgkin’s lymphoma, and lung adenocarcinoma indicate great variances within the same cancer types.^49^ These results are consistent with those observed from a study on about 10 000 cancer samples across 21 cancer types in The Cancer Genome Atlas.^50^ Both studies are based on 15 Buffa signatures instead of low-oxygen level as a direct indicator because it is not contained in the databases. Moreover, higher hypoxic signaling and hypoxia-associated genes tend to occur in tumors with higher diversity and are correlated with a poorer overall survival (OS) and progression-free survival (PFS).^51^ The intra- and inter-heterogeneity of hypoxic niche may explain the varied response to hypoxia-targeted therapy.

Hypoxic niche is also associated with increased mutational load of somatic variations and alterations of oncogenes and tumor suppressors, such as TP53, PTEN, and MYC.^49,52^

### Immune microenvironment: distinct in healthy tissues, primary lesions, and metastases

Turning cold cancers into hot ones is the goal of cancer immunotherapy nowadays. The former refers to treatment-naive cancers involving the inhibition of cytotoxic T cells, whereas the latter requires the generation and activation of cancer-associated T cells.^14,53,54^ TME encompasses malignant cells and T cells, as well as B cells, natural killer (NK) cells, tumor-associated macrophages (TAMs), myeloid-derived suppressor cells (MDSCs), mast cells, granulocytes, dendritic cells (DCs), tumor-associated neutrophils, cancer-associated fibroblasts (CAFs), adipocytes, vascular endothelial cells, and pericytes.^7,55^ The suppressive immune microenvironment helps cancer avoid immune destruction. The infiltration of immune cells in TME, located at the core or margin of cancer or the adjacent lymphoid organ or lymph nodes (also called tertiary lymphoid structures), is closely related to the prognosis in cancer^7,55–61^).

Cancer metastasis accounts for 90% cancer-related death for solid tumor.^62^ Undetectable micrometastases and unsatisfactory therapeutic responses are regarded as the two main problems causing high mortality through cancer metastasis. Pan et al.^63^ developed deep learning-enabled metastasis analysis in cleared tissue (DeepMACT) based on deep learning to detect micrometastatic foci and test the therapeutic targeting efficiency on mice with metastatic breast, lung, or pancreatic cancer. They demonstrated that antibody drugs are prone to be distributed to micrometastases within a close vicinity, suggesting probable participation of metastatic niche in determining targeting efficiency.^63^ TME plays a vital role in accomplishing metastatic cascade and is relatively distinct from normal tissues, primary tumors, and metastatic sites, especially when immune cells are involved.^7,64–66^ Malignant cells should escape immune surveillance, induce extracellular matrix (ECM) remolding, and achieve sufficient cell motility to migrate. CD4^+^CD25^+^ FOXP3^+^ regulatory T (T_reg_) cells, Ly6G^+^ neutrophils, MDSCs, and macrophages help build an immunosuppressive pre-metastatic niche, whereas TH1 CD4^+^ or CD8^+^ T cells, Ly6G^-^ neutrophils, and NK cells have the opposite functions.^67^ NK cells are downregulated in primary lung adenocarcinomas and lung cancer metastases compared with those in normal lung tissues.^68,69^ Fischer et al.^70^ compared the molecular profile of 88 melanoma brain metastases (MBMs) and 42 matched extracranial metastases with RNA sequencing (RNA-seq) data. They found that compared with extracranial metastasis, MBMs have less diverse T cell repertoire, fewer monocytes and DCs, comparable PAX5^+^ B cells and NK cells, more neutrophils, and greater activation of nervous system pathways.^70^

Friebel et al.^71^ revealed the leukocyte landscape for brain tumors through high-dimensional single-cell profiling. TME is distinct between gliomas and brain metastases: glioma TME has predominant tissue residence and reactive microglia, whereas brain metastasis TME possesses tissue-invading leukocytes. These findings are consistent with those of Klemm et al. (2020) obtained through flow cytometry, RNA sequencing, protein arrays, culture assays, and spatial tissue characterization. We used single-cell RNA-seq (scRNA-seq) to analyze the immune landscape of brain metastasis of lung adenocarcinoma.^72^ Analysis indicates that TAMs in brain metastasis inhibit T cell activation and infiltration, high levels of immune-suppressive macrophages, a unique alternative M2 activation signature, and a lack of conventional T cell co-stimulatory factors.^72^ The disparity of immune microenvironments among primary lesions and metastasis foci has provided an explanation for treatment failure in advanced patients with metastasis, especially brain metastasis. Besides, clinical trials often exclude patients with brain metastasis (except some clinical trials focusing on brain metastasis), thereby hindering the exploration of effective therapies for patients with brain metastasis. Insights into distinct immune microenvironments partially compensate for the situation, but more related clinical trials are still required.

### Metabolism microenvironment: focusing on lactate, reactive oxygen species (ROS), and lipid

Metabolic reprogramming (alteration in metabolism or nutrient supply) is one of the hallmarks of cancer. Cancer often shows increased metabolism of glucose, lipid, glutamine, and amino acids, lactate accumulation, and ROS addiction.^73–76^ Increased attention is being paid to the regulative effect of metabolism microenvironment on cancer cells. Organoid, Transwell, and tissue slice cultures have been applied to recapitulate cancer heterogeneity and metabolism.^18^

#### Lactate metabolism is involved in malignant and stromal cells

Normal cells tend to obtain energy through oxidative phosphorylation, and glycolysis is inhibited under normoxic condition. Cancer cells prefer enhanced glycolysis and elevated lactate metabolism even under normoxia, instead of oxidative phosphorylation.^20^ This phenomenon was first introduced by Warburg a century ago and named it the Warburg effect or aerobic glycolysis. Later on, Warburg stated that cancer cells prefer glycolysis over oxidative phosphorylation regardless of oxygen content.^77^ Since then, researchers have been interested in and focused on the following: (1) why cancer cells prefer aerobic glycolysis instead of oxidative phosphorylation, which can provide more energy; (2) how cancer cells utilize lactate metabolism; and (3) the potential ability of target lactate metabolism pathways to treat cancer. Although these issues remain unaddressed, we have achieved great progress over the past decades. Lactate is produced by malignant and immune cells in TME through the following: (1) conversion from glucose to pyruvate to lactate with the participation of lactate dehydrogenase; and (2) a series of processes starting from glutamine to glutamate to α-ketoglutarate, joining into the tricarboxylic acid cycle, and conversion of pyruvate with the participation of lactate dehydrogenase-A.^20^

Lactate has been regarded as a byproduct of metabolism, but emerging evidence shows that it may be a metabolite able to reprogram cancer cells and stromal cells in TME. It promotes macrophage polarization toward a pro-tumor and pro-inflammatory (M2-like) phenotype.^22,78^ The expression of Foxp3 suppresses Myc and glycolysis to promote the survival of T_reg_ in a high lactate microenvironment, thereby providing a supportive immunosuppressive microenvironment for cancer cells.^79^ Lactate promotes angiogenesis and the survival of hypoxic cells and induces an acidic microenvironment.^80^ Glutamine and glucose are two vital substrates for lactate metabolism, with glutamine being dominant when glucose is in deficit.^81^ Glutamine produces energy, carbon, and nitrogen for cancer cells and stromal cells, such as lymphocytes. Cancer cells uptake proteins, which can be degraded to glutamine that fuels the cancer cells through RAS-activated macropinocytosis.^82^ Several investigations have provided explicit and detailed introductions of glutamine metabolism in cancer^82–85^ revealed that breast cancer cells consume pyruvate in TME to cause collagen hydroxylation (not collagen synthesis) independently, induce ECM remodeling, and reprogram the lung metastatic niche. The pyruvate-mediated hydroxylation of collagen is driven by the activation of prolyl-4-hydroxylases regulated by α-ketoglutarate. In particular, pyruvate reportedly plays a role in the growth of spheroid rather than monolayer of breast cancer cells.^85^ Investigation of the products involved in lactate metabolism, including glucose, pyruvate, and glutamine can lead to the discovery of more potential therapeutic targets, as is being currently conducted.

#### ROS metabolism in TME

The elevation of ROS levels has been observed in cancers and is closely related with tumorigenesis, tumor immunity, and TME reprogramming.^86^ Under hypoxia, mitochondrial ROS are required in HIF stabilization,^87,88^ which may further induce autophagy and enhance tumorigenicity.^89^ Tumor cells and stromal cells in TME can produce ROS, whereas elevated ROS in local TME can affect the growth of cancer cells in return.^90^ Interestingly, as cancer cells evolve, they become tolerant to ROS accumulation and strike a balance with ROS, a phenomenon called ROS addiction.^73^ ROS in microenvironment can also affect the regulation of MDSCs, TAMs, CAFs, and T cells.^91–93^ Increased oxidative status in T_reg_ cells in ovarian cancer mice enhances its immunosuppressive function and resistance to programmed cell death protein 1 (PD-1)/programmed death-ligand 1 (PD-L1) therapy.^94^ Ligtenberg et al.^95^ demonstrated that decreased oxidative state facilitates better maintenance of T cell and antitumor activity with co-expressing catalase having chimeric antigen receptor T cell. Normalizing ROS metabolism in TME may assist immunotherapy to increase the effectiveness of their antitumor activity.

#### Lipid metabolism and formation of pre-metastatic niche

Lipids, including cholesterol, fatty acids, and triglycerides are materials for the generation of cancer cell membranes, post-translational modification of proteins, and energy for cancer cells.^75^ Lipid metabolism in TME regulates cancer growth, recurrence, and link diet with tumor. High cholesterol level is seen in TME and positively correlated with CD8^+^ T cell exhaustion.^51^ In a murine melanoma model, cholesterol increases endoplasmic reticulum stress, activates X-box binding protein 1 (XBP1), and upregulates PD-1 expression on CD8^+^ T cells, indicating that the combination of immunotherapy and cholesterol-reducing therapy may have great benefits.^96^ Strikingly, lipid metabolism promotes the formation of pre-metastatic niche in ovarian cancer. Hematogenous, lymphatic metastasis, and transcoelomic seeding are the three main routes for ovarian cancer metastasis. More than 80% ovarian cancer cells or spheroids have a tendency to migrate to the omentum, which is rich in adipocytes.^97^ Fatty acids are released during the lipolysis of adipocytes and are uptaken by cancer cells to fuel their growth. Previous studies have reported the ability of fatty acid-binding protein 4 (FABP4) to assist the solubilization, transport, and metabolism of fatty acids. It is upregulated in adipocytes and omental metastasis but primary ovarian cancer in patients and contributes to poor prognosis.^97,98^ The inhibition of FABP4 further induces lipid accumulation and inhibits adipocyte-mediated invasion.^97^ Lipid metabolism in TME may be a potential target for the prevention of cancer metastasis.

### Acidic niche: a result of hypoxia and lactate metabolism

Dysregulated or reversed pH has become a commonly recognized feature of cancer. Elevated intracellular pH (pH_i_) promotes cancer-cell survival, proliferation, migration, invasion, and glycolysis and inhibits apoptosis. Cancer cells tend to have a higher pH_i_ (7.4 vs. ~7.2) but a lower extracellular pH (pH_e_)(6.7–7.1 vs. ~7.4) compared with nonmalignant cells.^99^ It raises the acidic niche, manifesting as the acidosis of tumor and its microenvironment. Acidic niche cannot be separated from hypoxic niche and metabolism microenvironment, particularly the lactate metabolism microenvironment, because it is produced during lactate metabolism or by CO_2_ hydration.^24^ Hypoxia causes increased lactate production, proton accumulation in hypoxic niche, and tumor-cell adaptation through TME acidification.^100^ Lactate is exported by monocarboxylate transporter (MCT) 4, and is imported into cancer cells by MCT1 and co-transport of H^+^.^101^ During this process, tumor acidosis promotes tumor invasion and metastasis, and acidic niche has a synergic effect on lactate metabolism in providing a supportive microenvironment for cancer development. For the first time, lactate-based metabolic symbiosis has been recognized.^101^ Acidic niche increases oxidative phosphorylation, epithelial-mesenchymal transition, and invasiveness of melanoma cells.^102^ Similar results have been found in breast cancer and neuroblastoma cells.^103,104^ Oncogene activation (e.g., Ras and Myc) and inactivation of tumor suppressors (e.g., p53) also drive acidic niche generation.^100^ Acidosis has regulative effects on immune cells. For instance, low pH_e_ switch induces macrophage polarization toward M2 (alternatively activated) phenotype, activates neutrophil or DCs, and inhibits the cytotoxicity activity of TILs.^100,105^ Extracellular vesicle (EV) and exosome trafficking are also increased to transfer waste and excess acid under this situation.^106,107^ Acidic pH_e_ induces p-glycoprotein activation followed by p38 MAPK activation and causes resistance to daunorubicin. Meanwhile, vacuolar-type H^+^-ATPase (V-ATPase) is involved in intracellular alkalization. The inhibition of p38 MAPK or V-ATPase prevents metastasis and multidrug resistance,^100,108^ thereby confirming that maintaining pH homeostasis can serve as an approach against acidic niche.

### Innervated niche in TME

Awareness of the close relationship between neurology and cancer science has increased. Accordingly, Monje and other scientists have proposed a novel field called “cancer neuroscience” to better study how the nervous system communicates with cancer. Focus is given on electrochemical interactions, paracrine interactions, systemic neural-cancer interactions, and cancer-therapy effects on the nervous system.^109^ Moreover, increasing studies have confirmed that the nervous system participates in the development and metastasis of solid (e.g., prostate and brain) and hematological cancers.^28,31,110–112^

#### Innervated niche: an emerging specialized microenvironment focusing on the neural regulation of TME

The phenomenon of perineural invasion (PNI), representing cancer invasion in or around or penetrate nerve or route for cancer metastasis, is correlated with poor cancer prognosis.^31,110^ PNI has been reported in multiple cancers, such as those in the head and neck, pancreas, prostate, breast, colon, and ovaries.^28,30,110,113^ Amit et al.^114^ showed that oral cavity squamous cell carcinoma cancer cells with loss of TP53 can reprogram tumor-associated neurons to adrenergic phenotype depending on the signal transduction of EVs, which further promotes tumor progression. Injection of the nonselective adrenergic receptor blocker carvedilol to sensory fibers alleviates tumor proliferation and progression, indicating the significance of neural regulation in cancer.^114^ The communication mediated by EVs may partially explain the past failure of adrenergic nerve denervation in inhibiting tumor growth. We propose that the communications between nerve and cancer mediated by nerve-derived neurotransmitters or neuropeptides build a specialized localized microenvironment called “innervated niche”. Previous studies may refer to it as “perineural niche” or “neural regulation in TME”^112,115^ or “nerve microenvironment” in brain tumors.^116^ The innervated niche contains peripheral nerve (sympathetic, parasympathetic, or sensory), which has physical contact with cancer parenchyma or nerve located in the proximity of cancer that affects cancer cells. The innervation of cancer cells or stromal cells always relies on the release of neurotransmitters or neuropeptides, such as dopamine, catecholamine, and acetylcholine.^27^ The axonal microenvironment promotes the formation and development of schwannoma, a benign nerve sheath neoplasm.^116,117^ Gastric cancer secretes neurotrophins to promote nerve infiltration in TME.^117^ Active neurons promote the growth of high-grade gliomas via the neuroligin-3 (NLGN3)-activated PI3K-mTOR pathway.^118^ NLGN3 also upregulates synapse-related genes in glioma cells.^118^ In hematological cancers, the sympathetic nervous system innervates the egress of hematopoietic stem cells from the hematopoietic niche of bone marrow.^29,119^ Primary brain tumors including glioblastoma, schwannoma, astrocytomas, and brain metastasis are closely related with or even originated from neuron or nerve fibers; thus, their innervated niche is distinct from that for cancers of other organs.^116,117,120–123^ Accordingly, we suggest that the innervated niche be categorized as two main categories: intracranial and extracranial innervated niches.

#### Latest understanding of intracranial innervated niche

Venkataramani et al.^121^ and Venkatesh et al.^122^ verified that TME has a neural regulation of cancer and vice versa. Functional neurogliomal synapses generate AMPA receptor-mediated potassium currents,^121^ which can be amplified by gap junctions.^122^ This phenomenon occurs in about 5–10% of glioma cells, creates a direct electrochemical communication between glioblastoma cells and neuron, and promotes glioblastoma invasion and proliferation.^121,122^ Conversely, glioblastoma cells enhance neuronal excitability in TME, further altering currents dependent or independent on neurogliomal synapse formation. The depolarization of glioma cell membrane promotes cancer proliferation in xenografts.^122^ Tumor microtubes driven by growth-associated protein-43 also enhance astrocytoma progression and resistance.^120^ Approximately, 20% of cancer patients eventually develop brain metastasis, and the incidence of brain metastasis is even higher than that of a primary brain tumor.^65,124^ Zeng et al.^123^ have shown that brain metastasis also has a close crosstalk with its microenvironment. Gap junctions formed between breast/lung cancer and astrocytes assist cancer cells to transfer cGAMP to astrocytes, produce cytokines such as tumor necrosis factor and interferon-α, activate the STING pathway, and further stimulate NF-κB and STAT1 signaling in brain metastatic cells via paracrine; consequently, cancer growth and resistance to chemotherapy are promoted.^125,126^ In breast-to-brain metastasis, pseudo-tripartite synapses are generated between cancer cells and neurons. The activation of N-methyl-d-aspartate receptor-mediated colonization is triggered such that the reprogramed metastatic innervated niche becomes more supportive for cancer survival. Tumor co-opting nerves for survival and migration increase difficulty of cancer treatment but also serve as an entry point for therapies targeting the innervated niche.

### Mechanical microenvironment in TME

Mechanical microenvironment is another newly investigated specialized microenvironment.^32,34,36^ Its formation relies largely on intracellular components (vimentin, actin, and neurofilaments), extracellular components (collagen and fibrin), intercellular signaling (integrin), and stromal cells (fibroblasts).^127^ CAFs secrete matrix metalloproteinases (MMPs) including MMP2, MMP3, and MMP9 or activate yes-associated protein to promote ECM degradation and remodeling, epithelial-mesenchymal transition, and cancer-stem-cell stemness.^128–132^

Mechanical microenvironment influences oncogenes or tumor suppressors, cell morphology, cancer carcinogenesis, and therapeutic responses. Moreover, the mechanical stiffness of ECM is reported to accelerate glioblastoma cell progression.^36^ Cancer-produced ECM promotes the colonization of breast cancer lung metastasis through platelet recruitment via heat shock protein 47/type I collagen axis.^133^ Lu et al.^134^ and Leight et al.,^33^ provided informative discussions on the role of ECM in the modulation of cancer biology and treatment. HIF1 promotes lysyl oxidase production to enhance integrin signaling and increase the tumor-matrix stiffness.^135^ Park et al.^35^ confirmed that the mechanical microenvironment regulates glycolysis via tripartite motif-containing protein 21 and phosphofructo-1-kinase Isozyme C axis. They also demonstrated that the stiffness of cancer cells promotes their maintenance of fast metabolism, thereby further elucidating the heterogeneous specialized TME.^32^

## Crosstalk and nexus among specialized microenvironments

Awareness of crosstalk among TMEs has increased because of the advancements made in cancer research.

### TME reprogramming within hypoxic niche

Hypoxic niche occupies almost the entire tumor and external microenvironment. It influences cancer cells, reprograms other specialized microenvironments, and initiates a hypoxia-induced cascade. Particularly, immune, lactate and ROS metabolism microenvironment, and acidic niche are well-known top-affected TMEs. Hypoxia-induced VEGF expression is a typical product of the reshaping of immune microenvironment^96^ Horikawa et al.^136,137^ Sonveaux et al.^101^ demonstrated a phenomenon called “glycolytic switch” and “metabolic symbiosis”, and stated that oxidative cancer cells prefer utilizing lactate rather than glucose, in which MCT1 mediates a lactate exchange in tumors.^138^ Hypoxic cells consume glucose and produce lactate, which can diffuse based on the concentration gradient, whereas oxidative cancer cells uptake lactate via MCT1. After MCT1 inhibition, oxidative cancer cells change to utilize glucose rather than lactate. As the glucose gradient follows the gradient of oxygen supply, oxidative cancer cells are more likely to uptake glucose than hypoxic cells, causing their starvation for glucose and necrosis.^101^ Zhang et al.^139^ demonstrated that the hypoxia-lactate axis directly regulates gene expression through histone post-translational modification, which is called histone lactylation, thereby further inducing macrophage polarization toward being M2-like.^140^ This finding indicates a novel link between oncometabolites and histone code in hypoxic niche and highlights a counterbalancing homeostatic function for the hypoxia-lactate axis in regulating tumor immunity.

Hypoxic niche also has bidirectional communication with mechanical microenvironment mediated by CAFs. On one hand, they are involved in ECM remolding and the formation of hypoxic microenvironment.^141^ On the other hand, HIF-1 triggers the activation of PLOD2, P4HA1, and P4HA2 in response to hypoxia. CAFs become elongated and spindle shaped, secrete an increased amount of type I collagen, promote matrix adhesion and mesenchymal morphology, and produce ECM with increased stiffness and collagen fiber alignment, all of which support the invasion and migration of breast cancer cells.^142^ Recapitulating hypoxic niche while exploring the mechanisms may help fundamental researches obtain results close to clinical outcomes.

### Tumor-nerve-immunity cycle in TME

Recent studies have demonstrated a tumor-nerve-immunity cycle in TME, which mediates communication among cancer cells, immune microenvironment, and innervated niche, thereby disclosing the relationship among stress, immunity, and cancer.^143,144^ Nerve innervation in TME affects immune-cell recruitment and activation, cancer proliferation, metastasis, and response to immunotherapy.^28^ Released catecholamine may promote lymphocyte apoptosis and macrophage infiltration, inhibit NK cells and cytotoxic T cells, and thus facilitate tumor metastasis.^27,145,146^ High levels of norepinephrine suppress DC development and recruit MDSC to TME, causing tumor progression.^147^ The sympathetic nervous system guides the recruitment of macrophages or NK cells to tumor via β-adrenergic signaling.^148^ Elevated intratumoral IL-6 levels are seen in excised samples of stressed ovarian cancer patients compared with control patients.^28,149^

## Conventional drugs with new use: candidate for TME-targeting combination therapy

Numerous comprehensive reviews summarize the therapeutic strategies targeting cancer cells and TME.^18,28,72,150–156^ However, to our knowledge, drugs targeting TME are unsatisfactory. For example, treatment of BLZ945 (colony-stimulating factor-1 receptor inhibitor) inhibits macrophages, induces tumors regression, and prolongs survival in glioblastoma-bearing mice, whereas over 50% glioblastomas recur.^157,158^ Quail et al.^158^ demonstrated that the high relapse rate is due to acquired resistance and thus proposed the requirement of combination therapy. As TME is made up of various specialized microenvironments that overlap and communicate frequently with one another, targeting one specialized microenvironment may induce a series of changes in other specialized microenvironments and related pathways. Combination therapy targeting several specialized microenvironments may greatly benefit cancer treatment, along with intensive studies on the crosstalk within TME. The introduction of conventional drugs into the new application of targeting TME and treating cancer may enable its use in clinical practice and guide treatment choices.

### Drug repurposing in cancer

The appeal of drug repurposing in cancer is increasing for several reasons, such as the following: (1) it helps lower the cost (time, money, etc.) of developing a new drug; (2) it reduces the failure risk of clinical trials because those these drugs already have sufficient safety, toxicity, and pharmacological data; (3) drugs can be introduced to the market once sufficient antitumor effect is established.^159,160^ As reviewed in Pushpakom et al.,^159^ diverse approaches toward drug repurposing exist, including computational (signature matching, computational molecular docking, genome-wide association studies, pathway or network mapping, and retrospective clinical analysis) and experimental (binding assays to identify target interactions and phenotypic screening) approaches. To date, more than 200 noncancer drugs (cardiovascular, antibiotics, antiviral, antipsychotics, and antidepressants) have shown off-label antitumor effects. Among them, aspirin is the most frequently mentioned for drug repurposing in cancer considering its immunomodulatory effect on TME. Based on retrospective clinical data, aspirin was recommended to prevent colorectal cancer in 2015 (https://www.uspreventiveservicestaskforce.org/Page/Document/RecommendationStatementFinal/aspirin-to-prevent-cardiovascular-disease-and-cancer).

**Fig. 2:**
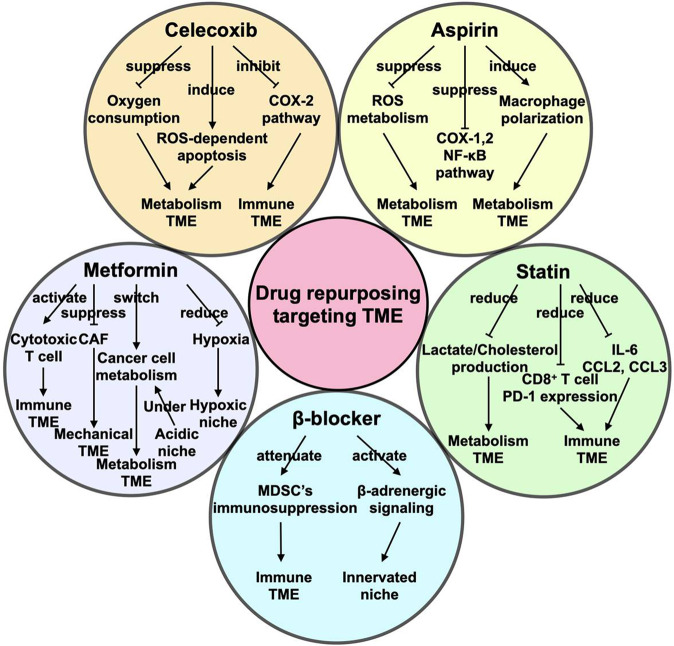
New life of old drugs in targeting TME. TME is regarded as a target for cancer therapy. Aspirin, celecoxib, β-adrenergic antagonist, metformin, and statin are five conventional drugs with antitumor capability that show potential use in combination therapy by targeting TME

Herein, we focus primarily on five promising drug-repurposing candidates, namely, aspirin, celecoxib, β-adrenergic antagonist, metformin, and statin (Fig. 2). These drugs have the following features: (1) they are well-known and commonly used in cardiovascular diseases, such as hypertension, diabetes, hyperlipidemia, and heart disease and have typical anti-inflammatory activity,^161,162^ and their retrospective electronic health records and pharmacological data are easily available; (2) they have been demonstrated to target TME and exert an antitumor effect in preclinical models; (3) they have been applied in abundant completed, recruiting, or registered clinical trials in cancer therapy alone or combined with standard treatment modalities (Table 1); and (4) they are required to treat the above-mentioned chronic comorbidities in cancer patients and may thus guide treatment selections in these patients.

**Table 1:** Clinical trials of conventional drugs in combination therapy against cancer

Conventional Drug	Combination Therapy	Targeting Cancer	ClinicalTrials.gov Identifier	Recruitment Status
Aspirin	Immunotherapy (atezolizumab, bevacizumab, pembrolizumab, avelumab, nivolumab, ipilimumab, lenalidomide)	Ovarian cancer, HNSCC, TNBC, solid adult tumor, multiple myeloma	NCT04188119	Not yet recruiting
			NCT02659384; NCT03245489; NCT03728179; NCT03428373	Recruiting
	Combination immunomodulatory cocktail (Vitamin D, aspirin, CTX and lansoprazole), pembrolizumab, radiation and curcumin	Cervical cancer, endometrial cancer, uterine cancer	NCT03192059	Recruiting
	Targeted therapy (osimertinib)	NSCLC	NCT03532698; NCT03543683; NCT04184921	Not yet recruiting
Celecoxib	Immunotherapy (nivolumab)	Metastatic cancer	NCT03864575	Not yet recruiting
	Targeted therapy (erlotinib, toripalimab, cetuximab, gefitinib, carboplatin, exemestane, depsipeptide)	Squamous cell carcinoma of oral cavity, colorectal cancer, breast cancer, NSCLC, HNSCC, ovarian cancer, pulmonary and pleural malignancies	NCT02748707	Active, not recruiting
			NCT03926338	Recruiting
			NCT00466505; NCT00068653; NCT00400374; NCT00072072; NCT00062101; NCT00499655; NCT01124435; NCT00201773; NCT00037817	Completed
	Chemotherapy (cisplatin, gemcitabine, docetaxel, paclitaxel and carboplatin, etoposide, irinotecan, capecitabine CTX, methotrexate, docetaxel, fluorouracil, vinblastine, 6-TG, temozolomide, CCNU, decitabine, DFMO, topotecan)	Pancreatic cancer, NSCLC, bladder cancer, TNBC, GBM, advanced cancer, colorectal cancer, cholangiocarcinoma, sarcoma, pulmonary and pleural malignancies, neuroblastoma, relapsed or progressive cancer	NCT02770378; NCT02030964	Active, not recruiting
			NCT02885974; NCT04081389; NCT03498326	Recruiting
			NCT00176813; NCT00030407; NCT00030420; NCT00062179; NCT00551005; NCT00101686; NCT00084721; NCT00551889; NCT02280694; NCT00320918; NCT00073866; NCT00064181; NCT00230399; NCT00085163; NCT00061893; NCT00047281; NCT00504660; NCT00037817; NCT00165451	Completed
	Radiation therapy	NSCLC	NCT00046839	Completed
	Targeted therapy and chemotherapy	Recurrent and/or refractory solid and CNS tumors	NCT02574728	Recruiting
	Chemoradiotherapy	Rectal cancer, cervix neoplasms, GBM, NSCLC, operable esophageal cancer	NCT00931203; NCT00336960; NCT00152828; NCT00112502; NCT00188565; NCT00346801; NCT00137852	Completed
	DC Vaccine, IFN alpha-2 and rintatolimod	Melanoma, peritoneal surface malignancies	NCT04093323	Not yet recruiting
			NCT02151448	Completed
	DC vaccine and cisplatin	Ovarian cancer	NCT02432378	Recruiting
	Gemcitabine and gene therapy (rAd-IFN)	Malignant pleural mesothelioma	NCT03710876	Recruiting
	Chemotherapy (carboplatin, gemcitabine) and 5-LOX inhibitor zileuton	Advanced NSCLC	NCT00070486	Completed
	Etoposide, CTX, thalidomide, fenofibrate	Relapsed or progressive cancer	NCT00357500	Completed
	ARTA and itraconazole	Multiple myeloma	NCT02401295	Completed
β-blocker	Immunotherapy (pembrolizumab)	Cutaneous melanoma	NCT03384836	Recruiting
	Chemotherapy (doxorubicin, vinorelbine, paclitaxel, nab-paclitaxel, doxorubicin, CTX)	EOC, primary peritoneal carcinoma, fallopian tube cancer, breast cancer, gastric cancer, Malignant soft tissue sarcoma, pediatric cancer	NCT02897986	Not yet recruiting
			NCT01847001	Active, not recruiting
			NCT04005365; NCT03108300	Recruiting
			NCT01308944	Completed
	NASIDs (etodolac)	Colorectal cancers; Pancreatic cancer	NCT03919461; NCT03838029	Recruiting
	Prednisone	Hemangioma	NCT01072045	Completed
	Relaxation/guided imagery audio intervention	Cervical cancer	NCT01902966	Active, not recruiting
Metformin	Immunotherapy (nivolumab, pembrolizumab, sintilimab)	NSCLC, SCLC, melanoma, solid tumors	NCT03048500; NCT03311308; NCT03874000; NCT03874000	Recruiting
			NCT02145559	Completed
	Targeted therapy (temsirolimus, vandetanib, sirolimus, lapatinib, erlotinib, irinotecan, gefitinib, sapanisertib, dabrafenib, trametinib, ritonavir, nelfinavir, toremifene, everolimus, letrozole, lanreotide)	Advanced cancers, kidney cancer, TNBC, colorectal cancer, NSCLC, advanced or metastatic relapsed or refractory cancers, lymphoma, melanoma, multiple myeloma or chronic lymphocytic leukemia, breast cancer, endometrial carcinoma, neuroendocrine tumors	NCT01529593; NCT02948283; NCT01797523	Active, not recruiting
			NCT01930864; NCT03017833; NCT02143050; NCT03829020; NCT02506790; NCT02823691	Recruiting
			NCT02495103; NCT01087983; NCT01650506; NCT01864681; NCT00659568	Completed
	Chemotherapy (docetaxel, carboplatin, paclitaxel, oxaliplatin, gemcitabine, vincristine, irinotecan, temozolomide, CTX, myocet, VPLD, R-CHOP, FDC)	Ovarian cancer, fallopian tube cancer, primary peritoneal cancer; esophageal squamous cell carcinoma, prostate cancer, pancreatic cancer, breast cancer, glioblastoma, pediatric tumor, childhood ALL, diffuse large-B-cell lymphoma	NCT03833466	Not yet recruiting
			NCT02336087; NCT01528046; NCT03200015	Active, not recruiting
			NCT02437812; NCT02122185; NCT04170465; NCT01929811; NCT03243851; NCT02506777	Recruiting
			NCT01796028; NCT01210911; NCT02312661; NCT02005419; NCT01666730; NCT01971034; NCT01442870; NCT01885013; NCT01324180	Completed
	Chemoradiotherapy	Cervix cancer, lung cancer, HNSCC, rectal cancer	NCT02115464; NCT02325401; NCT01430351	Active, not recruiting
			NCT02394652; NCT02949700	Recruiting
			NCT02437656	Completed
	Chemotherapy and targeted therapy	Endometrial cancer	NCT02755844	Active, not recruiting
	Antiandrogen (enzalutamide, bicalutamide)	Prostate cancer	NCT02339168	Active, not recruiting
			NCT02640534; NCT02614859	Recruiting
	Antibiotic (doxycycline)	Breast cancer, uterine cancer, HNSCC	NCT03076281	Active, not recruiting
			NCT02874430	Recruiting
	Chloroquine	IDH1/2-mutated solid tumors	NCT02496741	Completed
	Omega-3 fatty acids	Early stage breast cancer	NCT02278965	Active, not recruiting
	Vitamin C	Hepatocellular cancer, pancreatic cancer, gastric cancer, colorectal cancer	NCT04033107	Recruiting
Statin	Targeted therapy (sorafenib, erlotinib, anti-HER2 therapy, fulvestrant, aromatase Inhibitors)	hepatocellular carcinoma, squamous cell carcinoma, NSCLC, breast cancer	NCT03275376; NCT03324425; NCT02958852	Recruiting
			NCT00966472	Completed
	Chemotherapy (capecitabine, topotecan, CTX, zoledronate, bortezomib, bendamustin)	Rectal cancer, multiple myeloma, pediatric solid and CNS tumors, TNBC	NCT02569645; NCT02390843; NCT03358017	Recruiting
			NCT02161822; NCT00399867	Completed
	Chemoradiotherapy	GBM	NCT02029573	Completed
	Digoxin and enzalutamide	Prostate cancer	NCT04094519	Recruiting
	Ezetimibe	Prostate cancer	NCT02534376	Completed
Combination	Combination statin and celecoxib	Optico-chiasmatic gliomas	NCT02115074	Active, not recruiting
	Combination celecoxib, β-blocker, CTX, etoposide and vinblastin	Neuroblastoma	NCT02641314	Recruiting
	Combination aspirin, celecoxib and PD-1 antibody BAT1306	Colorectal cancer	NCT03638297	Recruiting
	Combination metformin and statin	Breast cancer	NCT01980823	Recruiting
	Combination statin, aspirin and dutasteride	Prostate cancer	NCT01428869	Completed

*NSCLC* non-small cell lung cancer, *SCLC* small cell lung cancer, *TNBC* triple negative breast cancer, *CTX* cyclophosphamide, *VPLD* vincristine, dexamethasone, doxorubicin, and PEG-asparaginase, *R-CHOP* rituximab, cyclophosphamide, doxorubicin, vincristine, prednisone, *FDC* fluoruracil, doxorubicin, cyclophosphamide, *ALL* acute lymphoblastic leukemia, *HNSCC* head and neck squamous cell carcinoma, *IDH* isocitrate dehydrogenase, *EOC* epithelial ovarian cancer, *NASIDs* non-steroidal anti-inflammatory drugs, *HER2* human epidermal growth factor receptor 2, *CNS* central nervous system, *GBM* glioblastoma, *6-TG* 6-thioguanine, *CCNU* lomustine, *DFMO* α-difluoromethylornithine, *DC* dendritic cell, *IFN* interferon, *5-LOX* 5-lipoxygenase, *PD-1* programmed death-1

### Aspirin

Aspirin, also called acetylsalicylic acid, is an extensively used anti-inflammatory agent, antiplatelet drug, and chemopreventive drug for inflammation-associated cancers. Regular use of aspirin is associated with lower risk of BRAF-wild-type colon cancer.^163^ It also exhibits antitumor activity in cancers including ovarian, gastric, and pancreatic through diverse mechanisms.^164–166^ Aspirin has an inhibitory effect on the cyclooxygenase (COX)-1 and COX-2 pathway to inhibit cancer proliferation, metastasis, and angiogenesis. The non-COX antitumor mechanism of aspirin includes the inhibition of the NF-κB or STAT3 pathway.^164^ Aspirin induces apoptosis by upregulating tumor necrosis factor-related apoptosis-inducing ligand and DR5 receptor or autophagy by inhibiting the mTOR pathway in cancer cells and xenografts.^167,168^ Numerous investigations have demonstrated that aspirin can target specialized TME.

#### Targeting the immune microenvironment

Previous studies have shown that platelet activation results in an immunosuppressive TME and spares cancer cells from immune surveillance, leading to their growth and migration.^139,169^ Considering that aspirin is one of the most commonly used antiplatelet drugs, Riesenberg et al.^169^ revealed its antitumor activity on the immune microenvironment and found its potential use in combination with PD-1 blockade. Similar results have been observed in breast cancer cells accompanied by decreased IL-8 secretion.^170^ Additionally, aspirin’s inhibition of the COX-1/thromboxane A_2_ pathway occurs through platelets, which involves the suppression of platelet aggregation on cancer cells, the recruitment of monocytes or macrophages, and the stepwise formation of premetastatic niche and tumor metastasis.^171^ Aspirin induces the polarization of macrophages toward M1 phenotype by increasing M1 marker expression while decreasing M2 marker expression and inhibiting cancer cell growth and migration in breast cancer cell lines.^172^ Aspirin also activates macrophage activity in eliminating therapy-generated tumor cell debris and inhibits macrophage-secreted proinflammatory cytokines.^173^ Aspirin further plays an immunomodulation role on other immune cells, including MDSCs and T_reg_ cells.^139^ These results suggest that aspirin regulates the immune microenvironment and is an attractive agent for combination therapy.

A recruiting phase II clinical trial (PRIMMO) has shown that aspirin, together with vitamin D, cyclophosphamide, and lansoprazole, forms an immunomodulatory cocktail and can be combined with immunotherapy pembrolizumab and radiotherapy to treat cervical and uterine cancers (NCT03192059) (Table 1). This accomplishment may provide additional data for aspirin’s effect on antitumor immunity.

#### Targeting the metabolism microenvironment

In 2002, aspirin was reported to inhibit cyclooxygenase, suppress oxidative stress and ROS metabolism, and inhibit ROS-mediated DNA damage.^174^ It has also been proposed to inhibit energy metabolism through AMPK activation and mTORC1 inhibition.^175^ Recently, Liu et al.^176^ revealed that aspirin regulates glucose metabolism to inhibit hepatoma cell proliferation.

In a study on the role of aspirin in reducing cancer risk, various doses, frequencies, and duration have been selected, such as the following: ≥975 mg/w for more than 5 years,^177^ ≥150 mg/d for more than 1 year,^178^ ≥325 mg/d for more than 5 years,^179^ 600 mg/d for a mean of 25 months,^180^ 100 mg/d for at least 104 d/y.^50^ Similarly, when assessing aspirin’s role in combination with other treatment modalities, these indices should also be carefully selected. Gastrointestinal bleeding is always a concern when using aspirin. Fortunately, data from patients with chronic viral hepatitis in Sweden show that low-dose aspirin does not significantly increase the risk of gastrointestinal bleeding and lowers the risk of hepatocellular carcinoma and liver-related mortality compared with non-users.^181^ However, the adverse effects and safety liabilities of aspirin should be carefully assessed under each condition.

### Celecoxib

Celecoxib is a nonsteroidal anti-inflammatory drug. Different from aspirin, celecoxib selectively inhibits COX-2 and is thus viewed as a COX-2 inhibitor. COX-2 is overexpressed in gastric, breast, and lung cancers, among others.^182^

#### Targeting the immune microenvironment

COX-2 induces immune escape and promotes cancer growth, suggesting the possible benefits of the COX-2 inhibitor celecoxib combined with immunotherapy.^183^ Over the past several years, a number of clinical trials on neoadjuvant immune checkpoint inhibitor (ICI) have been carried out in MMR-deficient (dMMR) and MMR-proficient (pMMR) colon cancer (NCT03026140). Compared with adjuvant ICI, neoadjuvant ICI is believed to stimulate a highly diverse immune response with the activation of TILs, especially in dMMR patients considering their higher mutational burden and more neoantigens.^184,185^ As celecoxib attenuates TIL suppression by inhibiting prostaglandin E2 (PGE_2_), it is introduced to neoadjuvant ICI (ipilimumab and nivolumab) in pMMR colon cancer patients hoping to achieve an improved clinical response.^186^

#### Targeting the metabolism microenvironment

Celecoxib suppresses oxygen consumption and induces ROS-dependent apoptosis in the metabolism microenvironment of metastatic melanoma and breast cancer, suggesting its potential as an enhancer for chemotherapy or radiotherapy.^187^

A large number of clinical trials focusing on the combination of celecoxib with targeted therapy, immunotherapy, chemotherapy, and radiation therapy have been completed or is recruiting. In a study on NVALT-4, celecoxib combined with docetaxel and carboplatin promotes survival in COX-2-high advanced NSCLC patients.^188^ However, most results are not encouraging. Altorki et al.^189^ showed that celecoxib introduction decreases prostaglandin E2 (PGE_2_), but COX-2 is induced by chemotherapy in NSCLC. Csiki et al.^190^ revealed that adding celecoxib to docetaxel inhibits COX-2 but does not improve the outcome of recurrent NSCLC patients. In a REMAGUS02 trial, a phase II randomized controlled trial, celecoxib combined with sequential neoadjuvant chemotherapy (NAC) (epirubicin + cyclophosphamide followed by docetaxel) shows poorer event-free survival and distant relapse-free survival and OS than the NAC group, particularly the prostaglandin-endoperoxide synthase 2 (PTGS2; also called COX-2)-low or estrogen receptor-negative group.^191^ The effects of celecoxib on breast cancer depend on the expression of PTGS2 and estrogen receptor status, as validated in PTGS2-low/high breast cancer cell lines. In vitro data show that adding celecoxib promotes cancer cell survival only in PTGS2-low cell lines.^191^ Another noteworthy phenomenon is that celecoxib may induce COX-2 expression in lung cancer cells. Expressed COX-2 protein could reportedly be transferred by exosomes to other cells such as monocytes and THP-1 cells, thereby increasing PGE_2_ and VEGF production.^192^ The reason behind the celecoxib-induced elevation of COX-2 expression remains unknown, but it confirms that celecoxib affects TME. Studies on the mechanism of the celecoxib and COX-2 pathway in cancer and TME can solve the complicated effect of celecoxib. Meanwhile, as more clinical trials reach completion, we may further understand of the positive and negative roles of celecoxib in cancer treatment.

### β-Adrenergic antagonist

As proposed by Tang et al.,^193^ β-adrenergic signaling is involved in eight hallmarks of cancer development, including proliferation, inhibition of tumor suppressors, cell death resistance, unlimited replication, angiogenesis, cancer invasion and metastasis, cellular energetics deregulation, and insensitive immune destruction.

#### Targeting the innervated niche

β-Adrenergic signaling is regarded as a significant pathway in innervated niche. Catecholamine-mediated β-adrenergic receptor activation causes the activation of β-adrenergic signaling, which plays a crucial role in cancer proliferation, invasion, metastasis, and angiogenesis.^148,193^ Catecholamine-mediated immunesuppression activity is even higher after surgery and likely attributed to psychological distress and pain. The cytotoxicity of NK cells also decreases. The use of β-adrenergic antagonist (β-blocker) or celecoxib days before surgery can improve immune surveillance.^194^ β-Blockers are other commonly used drugs for hypertension and arrhythmia. They can be categorized into selective β1-blocker (β1), nonselective β-blocker (β1 and β2), and α/β-blocker. In ovarian cancer, nonselective β-blockers prolong patients’ OS.^195^ The selective β1 blocker landiolol reduces lung cancer recurrence after operation.^196^ Additionally, β-blockers positively affect cancer patients by reducing their intrusive thoughts.^197^ Overall, the effects of β-blockers on TME and the whole organism indicate their potential application in future cancer treatments.

#### Targeting the immune microenvironment

β-Blockers attenuate MDSCs’ immunosuppression, promote the infiltration of T-lymphocytes and their cytotoxicity, and enhance immunotherapy efficacy.^143,198,199^ Chronic stress leads to MDSC accumulation and an immunosuppressive microenvironment; thus, tumor progression occurs through the activation of β2 adrenergic signaling; β2 blockers assist in attenuating MDSCs’ immunosuppression and sensitizing immunotherapy.^143,198^ Interestingly, β2 adrenergic signaling activation suppresses the glycolysis and oxidative phosphorylation of CD8^+^ T cells, suggesting that β2 blockers may be used to promote the cytotoxicity activity of CD8^+^ T cells.^200^

Apart from targeting TME and exerting an antitumor effect, β-blockers have been shown to prevent cancer therapy-induced complications such as cardiomyopathy and hypertension; thus, β-blockers are competitive in combinatorial therapy. Some studies have suggested that β-blockers prevent the ventricular dysfunction and cardiotoxicity induced by chemotherapy, and the reduction in the left ventricular ejection fraction mediated by β-blockers is <10%.^201,202^ To date, dexrazoxane is the only FDA-approved agent for anthracycline-induced cardiotoxicity prevention.^203^ The potential mechanism behind anthracycline-induced cardiotoxicity includes ROS production, iron-metabolism alteration, and Ca^2+^ channel change.^204^

Although VEGF inhibitor has been applied in various cancers due to its ability to reduce hypoxia-induced excessive angiogenesis,^205,206^ a great number of hypertension cases in cancer patients and increased arterial vascular events have been reported.^207^ Carvedilol may benefit the reversal of VEGF-induced hypertension due to its vasodilatory effect.^207^

However, the use of selective β1 (metoprolol) or β2 (bisoprolol) blockers may also promote VEGF-triggered microvessel sprouting, which does not apply to carvedilol (a nonselective β blocker).^208^ Some unsupportive data from several clinical trials have indicated that the use of β-blockers is correlated with higher overall mortality and recurrence rate, with the mechanism remaining undiscovered.^209,210^ The category of the β blocker is important to recognize and so pay attention could be paid to its side effects during the investigation.

### Metformin

Metformin is a well-known traditional antidiabetic drug, but its involvement in cancer, tuberculosis infection, and myotonic dystrophy has been increasingly observed.^211–213^ Metformin reportedly exerts antitumor activity in multiple cancers, such as gastric and thyroid cancers.^214,215^ More than 100 clinical trials have focused on the combination of metformin with chemotherapy, radiotherapy, immunotherapy, or targeted therapy in multiple cancers, including head and neck squamous cell carcinoma (HNSCC), gynecological cancer, myeloma, acute lymphocytic leukemia, and acute myeloid leukemia (Table 1). The use of metformin reportedly prolongs OS for high-grade glioma patients^216^ and decreases the incidence of colorectal cancer in type 2 diabetes patients in a dose-dependent manner.^217^

#### Targeting the immune microenvironment

Metformin can enhance immunotherapy or targeted therapy mostly due to its effect on the immune microenvironment. For example, adding metformin extends the PFS of patients with advanced pancreatic neuroendocrine tumors who are treated with everolimus and/or somatostatin analogs, lanreotide, or octreotide.^218^ Cha et al.^219^ demonstrated that metformin promotes the effect of anti-CTLA4 without toxicity in breast cancer cell lines, including triple-negative breast cancer (TNBC) by activating the tumor-infiltrating cytotoxic T cells and AMP-activated protein kinase (AMPK) pathway. AMPK activation further induces endoplasmic reticulum accumulation, endoplasmic reticulum-associated degradation, and decrease in PD-L1 level through PD-L1 phosphorylation and subsequent glycosylation.^219^ Tumor-infiltrating cytotoxic T cells are correlated with better prognosis in breast cancer.^220^ Meanwhile, increased TIL infiltration and PD-L1 level are also seen in patients with TNBC.^221^ In particular, some conventional drugs, including aspirin and atenolol can enhance the antitumor activity of metformin. In breast cancer, the AMPK pathway and complex I of the respiratory chain are two targets of metformin function. Adding aspirin activates the AMPK pathway and induces tumor cell apoptosis, whereas adding atenolol inhibits pericytes in breast cancer microenvironment; both increase the metformin-induced inhibition of complex I.^222^

#### Targeting the acidic niche

Metformin impairs the proliferation and colony formation of acidic melanoma cells and suppresses their metabolic adaptation in a dose-dependent manner (1–10 mM).^102^ As acidic niche is correlated with metabolic reprogramming, suppression of antitumor immunity, poor prognosis, and resistance to chemotherapy, metformin may help conquer chemotherapy-induced therapeutic resistance.^102^

#### Targeting the metabolism microenvironment

Preclinical models have revealed that metformin reprograms cancer cell metabolism.^102,223^ Metformin switches the metabolism of prostate cancer cells from being glucose dependent to being glutamine dependent^223^ and impairs metabolic adaptation and invasiveness of acidic melanoma cells.^102^ Metformin also influences lipid metabolism by suppressing adipocytes and inhibiting ovarian cancer growth and metastasis.^224^

#### Targeting the hypoxic niche microenvironment

Metformin inhibits the stabilization of HIF1α in mesothelial cells to impair ovarian cancer invasion.^225^ Metformin-mediated hypoxia reduction^205^ enhances the antitumor effect of PD-1 inhibitor in cancer cells.^226^ Hypoxia may predict who are irresponsive to metformin according to Sivalingam et al.^227^ They showed that metformin response decreases under hypoxic and hyperglycemia, and that cancer-cell metabolism is reprogrammed toward glycolysis under a hypoxic, high-glucose condition.

#### Targeting the mechanical niche microenvironment

Chen et al.^228^ showed that the effect of metformin on the survival of CAFs is relatively slight; in their study, the metformin concentration reaches 1 mM. They then co-cultured CAFs and cancer cells to explore metformin’s effect on CAFs and found that 0.2 mM metformin administered for 48 h significantly impairs the stimulatory effect of CAFs by upregulating the Calmodulin-like protein Calml3, thereby inhibiting the proliferation of gastric cancer cells.^228^ Metformin also downregulates the NF-kB signaling induced by CAFs to inhibit tumor progression.^229^

Metformin has been demonstrated to affect all proposed specialized microenvironments, suggesting this drug’s potential use in combination therapy. Interestingly, a recent study has shown that metformin displays its antitumor activity in mice only under hypoglycemia resulting from fasting dependent on the activation of PP2A-GSK3β-MCL-1 signaling.^230^ Together with Sivalingam et al.’s results (2020), metformin’s role in TME may be concluded to be significant under a normoxic, hypoglycemic condition. Thus, future studies should consider glucose and oxygen levels.

### Statin

Statin is an inhibitor of 3-hydroxy-3-methyl-glutaryl-CoA reductase and is used to downregulate the lipid level, thereby playing a role in the prevention of cardiovascular diseases.^231^ Statins can be classified as lipophilic (e.g., simvastatin and lovastatin) or hydrophilic (e.g., pravastatin and rosuvastatin). The intensive antitumor effects of lipophilic statins but not of hydrophilic ones in multiple cancers, such as lung, prostate, and breast cancers have been observed.^232–234^

#### Targeting the metabolism microenvironment

Statins affect the metabolism microenvironment. For instance, simvastatin induces metabolic reprogramming in HNSCC mice, reducing lactate production and promoting cancer sensitivity to MCT1 inhibitor. Simvastatin synergizes with MCT1 inhibitor AZD3965 to suppress cancer growth.^235^ Clendening et al.^236^ demonstrated that 7 of 16 multiple myeloma cell lines are sensitive to lovastatin-induced apoptosis. Lovastatin also influences DNA replication-associated genes, glycolysis, and cholesterol metabolism and exerts its antitumor effect in the ovarian high-grade serous carcinoma mevalonate pathway.^237^ However, Kutner et al.^238^ showed that statin use in end-stage patients (48.8% with cancer) with an estimated 7-month life expectancy is correlated with a shorter survival than those who stopped statin use (190 vs. 229 days) resulting from the reduced quality of life and low sense of well-being. No difference exists in the incidence of cardiovascular diseases (about 6%) between the two groups. These data indicate that statins may be stopped in terminal patients when considering the quality of life.^238,239^ Thus, the benefits and risks of statin use in cancer require great attention.

#### Targeting the immune microenvironment

Statins also reportedly target the immune microenvironment through cytokines or chemokines and immune checkpoints. In primary squamous lung cancer, statins break the communication between cancer cells and mesenchymal stromal cells (MSCs) by inhibiting CCL3 secreted by cancer cells and IL-6 and CCL2 produced by MSCs. This phenomenon suppresses the survival of lung cancer cells and indicates statin’s repurposing application in targeting the immune microenvironment.^240^ In a B16 melanoma lung metastatic model, statin lowers PD-1 expression in CD8^+^ T cells and effectively restores antitumor activity.^96^ These findings support the adjuvant role of statins combined with chemotherapy, radiotherapy, and targeted therapy (Table 1).

The above-mentioned drugs are only the tip of the iceberg in reference to all drugs that exert off-label antitumor effects. However, all these drugs target multiple specialized microenvironments and exhibit great potential in combination therapy, such as immunotherapy, chemotherapy, and targeted therapy. Our findings provide guidance for future drug repurposing, i.e., identifying TME-targeting drugs for combination therapy considering the great crosstalk among specialized microenvironments. Data from already completed clinical trials are not always supportive, which we believe is due to the poor understanding of the antitumor mechanisms behind each drug. Indeed, more preclinical experiments and clinical trials are needed to explore the right drug type, dose, frequency, duration, and suitable participator. Some issues requiring further clarification are as follows: (1) the advantages and disadvantages of using of β1, β2, and nonselective β blockers in cancer therapy; (2) the suitable dose of aspirin; and (3) the adverse effects of repurposed drugs and combination therapy considering the differences among diseases and patients. Challenges in drug repurposing for cancer treatment remain, including the lack of platforms for data analysis and high-throughput screening technologies.^159,161^ Dealing with these problems can promote and accelerate drug repurposing for cancer treatment.

## Discussion and outlook

TME comprises various specialized microenvironments that overlap and crosstalk with one another. In the above sections, we propose six specialized microenvironments in TME, focusing on their interaction with malignant cells and the crosstalk among them. We then provide a sophisticated landscape of TME. The understanding of TME as a compartment and a whole can provide directions for investigating combination therapy.

Hypoxic niche is more likely to cover the entire TME, as hypoxia is a constant status for a tumor and the microenvironment around it. All responses may not be isolated from the adaptation to the low-oxygen situation. Dewhirst et al.^241^ classified hypoxia in tumors into two categories: chronic hypoxia caused by hypoperfusion and acute hypoxia induced by change in perfusion. How does hypoxia proceed in actual solid tumors? How does each type of cell respond to hypoxia or tolerate it? These questions remain unanswered. Immune microenvironment is another key target for cancer treatment. Devalaraja et al.^242^ showed that tumor immune microenvironment facilitates the production of retinoic acid (RA) by tumor cells in sarcoma mice. RA promotes TAM differentiation and interferes with tumor immunity, and blocking the RA pathway induces a synergistic effect with anti-PD-1 therapy. The metabolism microenvironment, acidic niche, innervated niche, and mechanical microenvironment as emerging compartments of TME are under vigorous investigation.

In the classification proposed by Laplane et al.,^6^ microbiota is considered as an element of TOE beyond the TME. However, emerging evidence shows that apart from gut microbiota, microbiome can also be found in conjunctiva, mouth, nose, skin, and vagina and eventually become a part of TME. Thus, microbiome can directly interact with cancer, for instance, cervicovaginal microbiome for cervical or endometrial cancer and gut microbiome for gastrointestinal cancer.^243^ Microbiome is an emerging acellular component in TME that can influence carcinogenesis, genomic instability, and therapies and may even be involved in the process of metastases.^243,244^ Colorectal cancer cells can transport *Fusobacterium* to metastatic sites.^245^ *A. vaginae* and *Porphyromonas sp*. in the gynecologic tract are correlated with the occurrence of endometrial cancer for unknown reasons.^245^ Microbiota (*Bifidobacterium*) accumulated in colon adenocarcinoma promotes immunotherapy by activating STING signaling.^246^ Nejman et al.^247^ demonstrated that bacteria are present in cancer or immune cells, further confirming the presence of microbiomes in TME, although its role remains unknown. This area is largely unknown, and the definition of microbiome microenvironment requires consensus. Other acellular components such as EV/exosomes, cytokines, and chemokines in TME also play significant roles in cancer growth, metastasis, and drug resistance, as has been reviewed by Kalluri, LeBleu^1^ and Dassler-Plenker et al.^248^

With the aid of advanced research techniques such as scRNA-seq and organoids, exploring the compositions and functions of immune cells, as well as TME and its application potential, can be performed. Nearly 30% of metastatic sites may be undetected by traditional detection methods, such as computed tomography, magnetic resonance imaging, or bioluminescence imaging, whose results are read by an expert.^63^ DeepMACT has revolutionized the recognition and detection of micrometastasis. Meanwhile, the introduction of scRNA-seq, ATAC-seq, chromatin immunocleavage sequencing, or CUT&;RUN has expanded studies into single-cell scale over the past decade.^249^ Among them, scRNA-seq is viewed as one of the most helpful tools to analyze the cell subpopulation state. However, scRNA-seq alone cannot accurately reflect the interaction of the whole tumor with its microenvironment, especially in terms of spatial structure. Researchers have realized the limitation of scRNA-seq and attempted to solve this problem by integrating scRNA-seq with spatial transcriptomics (ST).^250^ ST was invented to obtain complete transcript data (spatial barcodes).^249,251–253^ The integration of scRNA-seq and multi-omics (transcriptomics, proteomics, metabonomics, and microbiome) may enhance our understanding of TME in a single-cell scale.

In this review, we propose that combination therapy may aid the new use of conventional drugs. As aforementioned, conventional drugs are highly available and have additional protective effects on other organs. Understanding their antitumor effect may guide the treatment of cancer patients with comorbidity. However, selecting the best treatment modality relies on the assessment of benefits and risks rather than focusing only on lowering risks and ignoring clinical efficacy. Combining conventional drugs with standard therapies is just one way for combination therapy or cancer therapy, but it may not always be the best one. Fortunately, we are moving forward. Corsello et al.^254^ have examined the antitumor activities of 4518 non-oncology drugs on 578 human cancer cell lines through the technique of profiling relative inhibition simultaneously in mixtures Their findings may be a revolutionary step in transitional cancer medicine and provide promising directions for repurposing drugs in cancer treatment.

We now understand that TME is a complicated ecosystem full of heterogeneity and can affect almost every aspect of cancer biology. It can be a target for cancer treatment, which still needs to be discussed and examined. However, the same as cancer, TME is an inevitable part of a patient. The close interactions of the TME with the whole organism and the effect of therapy on the whole body should be considered. As our understanding of TME is updated, we become more equipped to treat cancer.
